# Hormonal and inflammatory responses in prepubertal vs. pubertal male children following an acute free-weight resistance training session

**DOI:** 10.1007/s00421-024-05603-2

**Published:** 2024-09-11

**Authors:** Daniel Jansson, Elena Lundberg, Anna-Clara Rullander, Magnus Domellöf, Ann-Sofie Lindberg, Helena Andersson, Apostolos Theos

**Affiliations:** 1 Department of Community Medicine & Rehabilitation, Section of Sports Medicine, Umeå University, Linnaeus Väg 9, 90187 Umeå, Sweden; 2 Department of Clinical Sciences, Pediatrics, Umeå University, Umeå, Sweden; 3 Department of Nursing, Umeå University, Umeå, Sweden; 4 Department of Health, Education and Technology, Division of Health, Medicine and Rehabilitation, Luleå University of Technology, Luleå, Sweden

**Keywords:** Maturation, Endocrinology, GH, IGF-I, Growth, Fatigue

## Abstract

**Purpose:**

Examine the acute hormonal and cytokine responses to free-weight resistance training in trained prepubertal and pubertal male children.

**Methods:**

Prepubertal (n = 21; age 11.4 ± 1.1 years; Tanner I–II) and pubertal male children (n = 20; age 15.8 ± 0.7 years; Tanner III–V) conducted a moderate-intensity free-weight resistance training program to failure with venous blood sampling before (pre), immediately after (post) and during the recovery phase of the program (post-15,-30 min). Growth hormone (GH), insulin-like growth factor-I (IGF-I), cortisol, testosterone, IL-6, and TNF-α were analyzed in serum samples. Biological maturation was assessed according to the stages of the Tanner scale.

**Results:**

There was a significant time-by-group interaction in IGF-I response (*p* = 0.044; η^2^ = 0.209) and testosterone (*p* < 0.001; η^2^ = 0.508), indicating a greater change in the pubertal group compared to the prepubertal group. Both groups significantly increased post-exercise GH levels (*p* < 0.05). Only the prepuberal group significantly increased levels of IL-6 at all post-exercise time points (*p* < 0.05). Both groups showed a significant (*p* < 0.05) increase in TNF-α levels compared to resting levels.

**Conclusion:**

These data suggest that acute testosterone and IGF-I response following resistance training differ between trained prepubertal and pubertal male children. Moderate-intensity resistance training performed to failure may thus have different effects in trained prepubertal and pubertal male children, which should be considered when giving training advice.

**Trial registration:**

Clinical trials number: NCT05022992.

## Introduction

Biological maturation has important implications in youth sports (Malina et al. 2015). Mature youth perform better in athletic performance, such as strength, power, and endurance, than their less mature peers and have a more favorable anthropometry (larger body mass and stature) (Malina et al. 2015). Maturation also seems to affect neuromuscular fatigue, where the less mature youth experience less fatigue compared to their more mature peers (Souron et al. 2022). Further, maturation significantly affects the basal levels of hormones that are believed to be essential biomarkers affecting exercise response (Legerlotz et al. 2016). Higher mean serum levels of GH and IGF-I have been reported in fitter adolescents versus less fit (Eliakim et al. 1996), suggesting a link between anabolic hormones and fitness. The onset of puberty induces rapid endocrine secretion of sex steroids (SHBG, testosterone) and GH-IGF-I (growth hormone–insulin-like growth factor I) axis hormones (Boisseau and Delamarche 2000; Löfqvist et al. 2001; Riddell 2008), suggesting that maturation may affect the endocrine response.

Previous research has shown that substantial adaptation of the GH-IGF-I axis to physical activity and exercise exists in youth (Eliakim et al. 1996; Jansson et al. 2022). After acute exercise, GH notably increases following exercise training, while IGF-I has modest changes (Nemet et al. 2002). Surprisingly, long-term training studies have not shown similar high anabolic activation in youth (Jansson et al. 2022). Other researchers (Nemet et al. 2002) have suggested that proinflammatory cytokines such as IL-6 and TNF-α are partly responsible for the reductions in anabolic mediators typically seen after an initial training program in youth (Nemet et al. 2002). This hypothesis has been derived from studies showing significant post-exercise increases in proinflammatory cytokines such as IL-6, TNF-α, and IL-1β while anabolic mediators such as IGF-I decrease (Nemet et al. 2002). The results have been replicated in another study on children (Nemet et al. 2003) with similar results, typically following endurance-type activities but not resistance training.

In the realm of resistance training, research on the GH-IGF-I axis has almost exclusively been carried out in adults (Kraemer and Ratamess 2005). Falk and Eliakim (2014) suggested that a reason for the scarce data on similar studies on children may be because anabolic hormones such as GH are traditionally associated with hypertrophy, which is rarely observable in children. The limited studies conducted in the field suggest that levels of GH seem to acutely increase following resistance exercise in both adolescents (Pullinen et al. 2002) and children (Rubin et al. 2014) and no changes in some cases (Pullinen et al. 2011). Similar acute hormonal responses of the GH-IGF-I axis have been reported in adults and 9-year-old children (Rubin et al. 2014). While the adults differed from children in some cases, the authors speculated that the difference was mainly due to activated lean mass and not maturation (Rubin et al. 2014).

Scarce evidence exists on the effects of biological maturation on neuromuscular performance (Souron et al. 2022) and the acute hormonal response to resistance training (Falk and Eliakim 2014; Sekine and Hirose 2022). The hormonal response to resistance training is associated with neuromuscular performance. Heavy muscular work leads to a momentary decrease in strength and voluntary maximal neural activation of the loaded muscles (Ahtiainen et al. 2003). Although children resist fatigue better than adults, current literature has rarely considered the comparison of adolescents versus children for the evaluation of fatigue (Souron et al. 2022). Furthermore, the limited evidence suggests that the acute hormonal response to resistance training may vary depending on maturation (Sekine and Hirose 2022). This has been evident by a greater acute testosterone response in more mature children following a body-weight resistance training program, while no difference was observed in GH or cortisol response (Sekine and Hirose 2022). Hormones such as GH and testosterone, and puberty-related hormone-binding proteins, such as SHBG, are affected by growth and puberty and regulate the relationship between fat and lean body mass in children (Tsolakis et al. 2003). Although GH and SHBG are influenced by pubertal development, they have been reported to remain unchanged after long-term resistance training in both prepubertal and pubertal males (Falk and Eliakim 2014; Tsolakis and Messinis 2020), whether similar effects are evident after an acute bout of resistance training is less clear. Additionally, studies using loaded resistance training in children and adolescents are lacking and may be superior since exercise intensity is a critical determinant of the acute hormonal response (Kraemer and Ratamess 2005; Kraemer et al. 2020).

Thus, in this study, we investigated the acute hormonal and cytokine responses in trained prepubertal and pubertal male children following acute free-weight resistance training. Comparing the responses of these two groups may help improve our understanding of the role of maturation in the exercise-induced hormonal and inflammatory profile of trained male children.

## Methods

### Study design

In a three-day study design, all tests, physical sessions, and medical examinations were conducted in the UMEX laboratory at Umeå University, Sweden. Before the main trial, participants underwent two familiarization sessions to acquaint themselves with the equipment and procedure. These sessions also included physical tests to establish the resistance training intensity (10 RM) for the two exercises (leg press and bench press) used in the primary experimental trial.

Each session began with a warm-up consisting of 5 min of cycling on a cycle ergometer (60 W) and a 5–10 min whole-body dynamic warm-up (squats, lunges, leg swing, push-ups). During the first session, participants familiarized themselves with the training protocol (10 RM leg press and bench press). This session was an introductory session before the primary trial to ensure the participants could safely perform the exercises. Participants were taught the proper techniques for the strength exercises (leg press and bench press), and any questions they had were answered. They also completed a health assessment form (including questions about training experience, medication, and overall health status) and underwent anthropometrical tests (lean leg volume, body mass, height, and sitting height) and a submaximal cycle ergometer test. To establish their blood pressure levels, after 5 min of seated rest, systolic (SBP) and diastolic blood pressure (DBP) were measured.

During the second session, the participants conducted tests to determine their 10 RM bench press and 10 RM leg press. In the third and final session, blood samples were collected at rest (pre) and after a resistance training session (post 0 min, post 15 min, and post 30 min). The blood sampling session was conducted at a similar time of day (16.00–18.00) to minimize the impact of the circadian rhythm (Hackney and Viru 2008). While most previous pediatric trials examining the effects of exercise on hormones have measured levels during the morning after overnight fasting, our trial was conducted to reflect real-life training situations that typically occur during the day or evening, as supported by previous research (Klentrou et al. 2016).

### Outcomes

The primary outcomes were changes in circulating growth hormone (GH) concentrations and insulin-like growth factor-I (IGF-I) pre- to post-training. The secondary outcomes were cortisol, testosterone, interleukin-6 (IL-6), TNF-α, and sex hormone-binding globulin (SHBG).

### Participants and recruitment

Participants were recruited to the study in the spring of 2022 using public announcements, posters displayed around the city area, social media, and invitations sent out to sports clubs. Inclusion criteria were: healthy males (age: 10–12 yrs and 14–16 yrs) physically active in various sports such as soccer, floorball, and volleyball and had no/or little experience in resistance training. Participants were divided into two groups based on the biological maturity range (prepubertal (Tanner level I–II)- pubertal (Tanner level III–V). Exclusion criteria were chronic diseases or injuries. Participants were instructed to avoid strenuous physical activity the day before testing and not to eat heavy meals two hours prior to the tests. Written informed consent was obtained from all participants and their caregiver. Ethical approval was obtained from the Swedish Ethical Review Authority (Nr: 2020-03179), and the study protocol was pre-registered at ClinicalTrials.gov (NCT05022992).

#### Participant flow

**Fig. 1:**
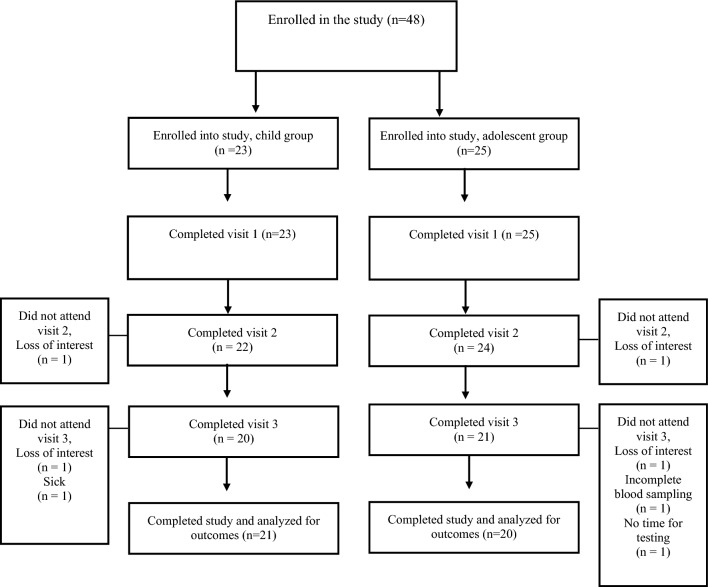
Participant flow chart of the 3-day testing sessions

A total of 48 participants, comprising of 23 prepubertal and 25 pubertal, were initially enrolled in the study (Fig. 1). Four participants later withdrew from the study, citing a loss of interest. One participant fell ill before the final visit and was unable to undergo follow-up tests. Additionally, one participant had to withdraw from the study due to time constraints. Finally, one participant attended all scheduled visits but had to cancel the last session because the nurse were unable to take blood sample. The remaining participants successfully completed all study visits and tests. Thus, in total, 41 healthy and physically active males, with 21 in the prepubertal group (mean age: 11.4 ± 1.1 years, Tanner I–II) and 20 in the pubertal group (mean age: 15.8 ± 0.7 years, Tanner III–V) completed all sessions, as shown in Table 1. Due to the varying availability of analyzed hormones/cytokines, total N varied depending on each cytokine/hormone (range 16–20/group).

**Table 1:** Participant and physical characteristics of the study population

Outcomes	Prepubertal (n = 21)	Pubertal (n = 20)	*p*
	Mean (SD)	Mean (SD)	
Age (yrs)	11.4 (1.1)	15.8 (0.7)	< 0.001
Hb rest (g/L^−1^)	115.1 (39.2)	144 (9.0)	0.004
Body mass (kg)	42.1 (7.9)	66.9 (8.1)	<0.001
Body mass SDS^a^	0.71 (1.45)	0.57 (0.9)	0.725
Height (cm)	150.6 (8.8)	179 (7.0)	< 0.001
Height SDS^a^	0.51 (0.98)	0.61 (1.2)	0.778
BMI (kg/m^2^)^b^	18.6 (2.23)	18.3 (2.3)	0.699
Resting SBP (mm Hg)	111.8 (5.5)	115.2 (9.2)	0.159
Resting DBP (mm Hg)	69.3 (4.3)	72.4 (5.9)	0.071
Fat-free mass (kg)^c^	33.3 (5.3)	58.0 (6.6)	< 0.001
Sum of two skinfolds (mm)^c^	23.1 (8.9)	17.6 (4.3)	0.016
Percent body fat (%)^c^	21.4 (7.2)	13.2 (4.0)	< 0.001
Lean leg volume (L)	3.4 (1.2)	7.3 (0.9)	< 0.001
Leg volume (L)	5.0 (1.6)	9.0 (1.1)	< 0.001
Training experience (y)	5.2 (1.3)	8.2 (2.5)	< 0.001
Training volume (h/week)	4.4 (1.9)	7.6 (2.9)	< 0.001
Leg press, 10 RM (kg)	50.0 (16.2)	103.5 (24.4)	< 0.001
Bench press, 10 RM (kg)	17.4 (4.2)	41.0 (10.1)	< 0.001
Estimated VO_2_max (ml/kg/min)	54.1 (8.4)	63.2 (7.7)	< 0.001

Values presented as mean and standard deviations (SD)

*BMI* body mass index, *SDS* standard deviation score, *Hb* hemoglobin, *SBP* resting systolic blood pressure, *DBP* diastolic blood pressure

^a^Swedish population-based longitudinal reference values from birth to 18 years of age for height, weight, and head circumference (Wikland et al. 2002)

^b^BMI reference value (mean and SD) for Swedish children (Karlberg et al. 2001)

^c^Estimated from youth-specific equations (Slaughter et al. 1988)

### Maturation

An experienced physician performed a clinical examination, and puberty was assessed according to the Tanner stage (Marshall and Tanner 1970) and the Prader orchidometer (Prader 1966).

### Anthropometrical assessments

To describe the participants’ characteristics, basal anthropometrical variables were tested. All participants wore shorts and t-shirts. Height (cm) and sitting height (cm) were measured using a wall-mounted stadiometer (SECA, UK) to the nearest 0.1 cm. Body mass was measured using a digital scale (SECA, UK). Lean leg volume (LLV*)* was determined by using a previously validated anthropometric model by Jones and Pearson (1969). In short, we measured the circumference of seven sites of the leg and height while the participant was standing (Jones and Pearson 1969). Skinfolds of the leg at four sites were measured. Lean leg volume was determined by subtracting the subcutaneous fat volume from the total leg volume. Skifolds were measured in duplicate at two sites, triceps and subscapular, on the right side of the body, using a caliper (Harpenden, British Indicators Ltd, St Albans, Herts). Body fat percentage was determined according to youth-specific equations by Slaughter et al. (1988). The same researcher conducted all anthropometrical measurements.

### Aerobic fitness

Participants conducted a validated submaximal cycle test, as previously described (Björkman et al. 2018), to estimate their basal cardiorespiratory fitness. VO_2_ max was estimated using sex- and age-specific equation (Björkman et al. 2016).

### Muscular strength

Upper- and lower-body maximal strength was estimated by performing a 10 RM leg press and bench press test. The leg press was conducted in a horizontal leg press machine (Agaton Fitness AB, Boden, Sweden), while the bench press test was conducted with a barbell (Eleiko, Halmstad, Sweden). For 10RM tests, participants performed a set of 8–12 repetitions with light loads, ten reps with relatively light loads, 10 reps with moderately heavy loads, and 10RM attempts until failure. An increase in loads of a minimum of 0.5 kg weight increase followed successful 10RM attempts. After each exercise set, the subjective rating of perceived exertion was assessed using a 10-point scale adapted for children (Eston et al. 2009) to assist in adjusting the load. In general, 10RM was determined within four to six attempts.

### Main trial

Blood samples were taken before and after a moderate-intensity resistance training session. The acute, moderate-intensity exercise protocol was developed in line with youth resistance training guidelines (Faigenbaum et al. 2009; Lloyd et al. 2014). The training session comprised four sets of predetermined 10RM for leg press and bench press conducted until failure. The exercise was chosen due to the involvement of large muscle mass, which is generally considered important for acute anabolic hormone secretion (Kraemer and Ratamess 2005). The inter-set rest interval was set to 1 min, and a 3-min rest interval was provided between exercises. The training program was approximately 20 min, supervised by an experienced strength coach and participants were well-familiarized with the protocol. Participants’ heart rate was monitored during the training session.

#### Fatigue

The fatigue index (FI%) for each exercise was calculated based on the successful repetitions in each set for the predetermined load using the following equation: (set 1–set 4) / set 1 * 100, as previously described (Weinstein et al. 2018).

#### Blood analysis

An intravenous (IV) catheter was inserted into an antecubital or dorsum-of-hand vein. Blood samples (5 ml) were taken before training, after, and at 15 and 30-min post-recovery. The blood samples were allowed to clot at room temperature (1 h) and centrifuged at 1500 g at 4 °C for 15 min (Allegra X-22R, Beckman and Coulter, Brea, CA, USA), directly afterward, serum was extracted, and aliquots were stored in 0.5-ml polyethylene tubes at −80 °C until assayed. A small amount, 50–70 μL blood was used for analyzing hemoglobin levels (HemoCue, Ängelholm, Sweden).

#### Hormone assay

All samples were assayed in duplicate, and samples with CV% > 10% (resp. 15% for TNF-α and IL-6) were re-assayed. IGF-I, IL-6, and TNF-α were analyzed with high-sensitivity bead-based multiplex assays using the Luminex technology (MILLIPLEX map kit, Millipore). SHBG, cortisol, testosterone, and GH were measured by enzyme-linked immunoassay (ELISA, R&D Systems Inc., Minneapolis, US). The free androgen index was calculated from the ratio of testosterone/SHBG (Kraemer and Ratamess 2005) and was determined to account for the change in the bioavailability of testosterone. The inter-assay of variations were as follows: testosterone: 5.6% (n = 7), cortisol: 14.4% (n = 7), SHBG: 7.0% (n = 6), IGF-I: 9.0% (n = 5), GH = 6.0% (n = 6), IL-6: 5.9% (n = 5), TNF-α: 2.2% (n = 5). The sensitivity according to the protocols was: IGF-I: 19 pg/mL, IL-6: 2.95 pg/mL, TNF-α: 6.23 pg/mL, testosterone: 0.041 ng/mL, cortisol: 0.111 ng/mL, SHBG: 0.012 nmol/L, and GH: 7.18 pg/mL, respectively.

### Statistical analysis

A post hoc power analysis was performed with a significance level of 0.05 and a power of 80%. The analysis showed that a sample size of 38 subjects is sufficient to find a 12%, 29%, and 16% difference in testosterone, GH, and IGF-I change (from baseline to post-exercise) between the prepubertal and pubertal groups. Descriptive statistics were presented as mean ± SD, and a standard Student unpaired T-test was used to compare baseline measurements between groups. Shapiro-Wilks test and visual inspections of histograms were used to assess normality. GH, IL-6, and TNF-α were not normally distributed and hence were log-transformed before performing statistical analyses (data are presented in their original form). A two-way repeated ANOVA design was employed with one between factor (group: prepubertal vs. pubertal) and one within factor (time: blood samples) to compare prepubertal and pubertal males hormonal/cytokine response pre-and post-training. Post-hoc comparisons were conducted using the least significance difference method. We also reported effect size according to partial eta squared (η^2^), which was considered small (~ 0.01), moderate (~ 0.06), or large (≥ 0.14) (Cohen 1988). In addition, Cohen’s d values were calculated for significant acute effects of each hormone to quantify the effect size. Cohen’s d values were interpreted as small (0.2), medium (0.5), and large (0.8) (Cohen 1988). The significance level was set at α = 0.05. The statistical analysis was performed using the SPSS statistical package (SPSS, v. 22, Chicago, IL).

## Results

### Participant characteristics of the study population

The clinical physical characteristics of the study population are presented in Table 1. Compared to prepubertal, the pubertal male children were significantly taller, with greater mass and fat-free mass (*p* < 0.001). In addition, pubertal male children were stronger in all muscular strength tests and had longer training experience and greater weekly training volume (*p* < 0.001).

### GH-IGF-I

**Fig. 2:**
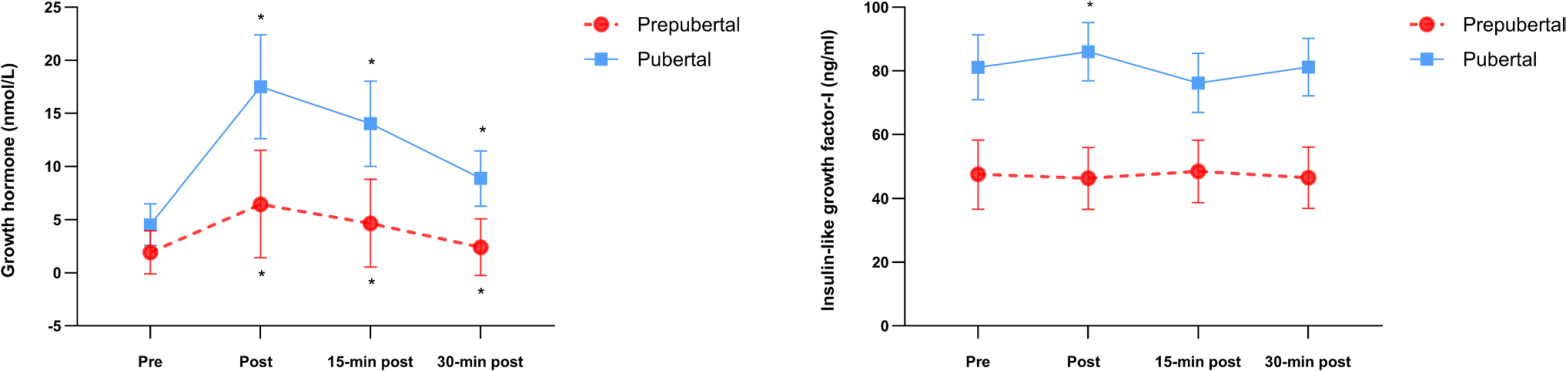
Mean GH and IGF-I response to a moderate intensity resistance training session in pubertal and prepubertal male children. Values are presented as mean and 95% confidence intervals (CI). Growth hormone: significant group-by-time interaction (p = 0.011). # IGF-I: significant group by time interaction (*p* = 0.044). * within-group differences greater than baseline, *p* < 0.01

Levels of IGF-I (nmol/l) were consistently higher across all time points in the pubertal group compared to the prepubertal group (Table 2**, **Fig. 2). There was no significant main effect observed for time (*p* = 0.309, η^2^ = 0.099), but a notable main effect for group (*p* < 0.001, η^2^ = 0.444), along with a significant time-by-group interaction effect (*p* = 0.044; η^2^ = 0.209). Pairwise comparisons revealed no significant acute impact of the resistance training protocol on IGF-I levels at different time points in the prepubertal group (*p* > 0.05). However, there was a significant increase in IGF-I immediately following the resistance training session in the pubertal group compared to resting levels (pre vs. post; mean difference: 4.8 ng/mL; *p* = 0.049; d = 0.57).

**Table 2:** Serum hormone and cytokine concentrations before (pre), after (post), and during the recovery (15-min and 30-min post-exercise) of a moderate-intensity resistance training session in prepubertal and pubertal male children

	N	Pre	Post	15-min post	30-min post	Time	Time x group
		Mean (SD)	Mean (SD)	Mean (SD)	Mean (SD)	Effect	Interaction
Hormones							
GH (nmol/L)^b^							
Prepubertal	19	0.4 (0.1–2.6)	6.8 (2.0–9.4)	4.1 (2.6–5.4)	2.1 (1.2–3.2)	*p* < 0.001	*p* = 0.011
Pubertal	19	2.0 (0.5–7.6)	12.4 (8.2–22.8)	11.1 (7.3–15.5)	7.3 (4.9–9.9)		
IGF-I (ng/mL)							
Prepubertal	18	47.5 (16.1)	46.3 (16.4)	48.5 (16.9)	46.5 (18.0)	*p* = 0.309	*p* = 0.044
Pubertal	20	81.1 (27.0)	86.0^a^ (23.0)	76.2 (23.1)	81.2 (21.5)		
Testosterone (nmol/L)							
Prepubertal	19	4.8 (2.4)	4.5 (2.5)	4.3 (2.3)	4.4 (2.4)	*p* < 0.001	*p* < 0.001
Pubertal	19	23.3 (6.4)	23.0 (7.3)	20.6^a^ (6.1)	19.2^a^ (6.1)		
Cortisol (nmol/L)							
Prepubertal	17	151.1 (46.2)	121.3 (40.8)	126.3 (63.7)	124.1 (60.1)	*p* = 0.032	*p* = 0.212
Pubertal	20	180.1 (70.9)	182.1 (94.5)	207.8 (99.9)	195.0 (92.1)		
SHBG (nmol/L)							
Prepubertal	19	76.5 (30.4)	79.0 (27.8)	76.2 (29.1)	76.2 (30.4)	*p* = 0.011	*p* = 0.001
Pubertal	20	36.2 (11.7)	36.4 (10.7)	35.4 (11.0)	34.9 (11.6)		
Free androgen index							
Prepubertal	18	6.7 (3.4)	6.4(3.4)	6.3 (3.7)	6.4 (3.5)	*p* < 0.001	*p* < 0.001
Pubertal	19	70.4 (30.9)	69.0 (30.3)	63.5^a^ (27.7)	60.6^a^ (27.4)		
Cytokines							
IL-6 (pg/mL)^b^							
Prepubertal	16	1.7 (0.7–4.5)	1.7 (0.8–4.4)	2.2^a^ (0.8–5.5)	2.3^a^ (1.1–5.6)	*p* = 0.036	*p* = 0.016
Pubertal	20	10.3 (4.5–20.1)	9.9 (4.7–21.0)	9.1 (4.5–21.3)	8.1 (4.4–21.0)		
TNF-α (pg/mL)^b^							
Prepubertal	18	10.3 (7.9–11.8)	12.2 (9.4–14.8)	10.4 (8.9–13.3)	12.2 (9.7–13.4)	*p* = 0.002	*p* = 0.102
Pubertal	20	12.3 (10.9–15.5)	14.4 (11.4–18.2)	14.2 (12.3–17.4)	13.4 (11.4–16.9)		

Data are presented by mean ± standard deviations (SD)

*GH* growth hormone, *IGF-I* insulin-like growth factor I, *IL-6* interleukin 6, *TNF-α* tumor necrosis factor-alpha, *SHBG* sex hormone-binding globulin

^a^Significant difference between prepubertal and pubertal groups, *p* < 0.05

^b^GH, IL-6 and TNF-α are logtransformed and data presented by median and 25–75% percentiles

GH (nmol/l) response was significantly higher at all time points in the pubertal group vs. the prepubertal group, except for the baseline levels (*p* = 0.072; Fig. 2). There was a significant main effect for time (*p* < 0.001, *η*^2^ = 0.923), group (*p* < 0.001, *η*^2^ = 0.373) and a significant time-by-group interaction (*p* = 0.011, *η*^2^ = 0.281). Pairwise comparisons revealed a significant acute impact of the resistance training protocol on GH levels at all post-exercise measurements (all, *p* < 0.05) for both the prepubertal and pubertal groups.

### IL-6 and TNF-α

For IL-6, there were a significant effect of time (*p* = 0.036; *η*^2^ = 0.232), group (*p* = 0.018; *η*^2^ = 0.154) and interaction (*p* = 0.016; *η*^2^ = 0.271). Pairwise comparisons showed that the prepubertal group significantly increased IL-6 levels at post-exercise (*p* = 0.006; d = 0.44), 15-min post-exercise (*p* = 0.007; d = 0.52), and 30-min post-exercise (*p* < 0.001; d = 1.04) as compared to resting values (Table 2). No acute effect was observed for the pubertal group (*p* > 0.05).

There was a significant main effect for time (*p* = 0.002, *η*^2^ = 0.350) and group effect (*p* = 0.005, *η*^2^ = 0.196) but no significant time-by-group interaction effect (*p* = 0.102; *η*^2^ = 0.165). Concentrations of TNF-α increased significantly at post-exercise (*p* < 0.001), 15-min post-exercise (*p* = 0.004), and 30-min post-exercise (*p* = 0.002) as compared to resting values for both groups (Table 2).

### Testosterone, cortisol and SHBG

Testosterone levels (nmol/l), were significantly higher at all time points in the pubertal group vs. the prepubertal group (Table 2). There was a significant main effect for time (*p* < 0.001, *η*^2^ = 0.577) and group (*p* < 0.001, *η*^2^ = 0.773). Moreover, a significant time-by-group interaction effect was observed (*p* < 0.001; η^2^ = 0.508), suggesting differing patterns of change between the groups across time points. Pairwise comparisons revealed no significant acute impact of the resistance training protocol on testosterone levels at any time point for the prepubertal group (*p* > 0.05). However, in the pubertal group, testosterone levels experienced a significant decrease following the resistance training session, evident at 15 min and 30 min post-exercise compared to resting levels (pre- vs. post-15 min; mean difference −2.65 nmol/L, *p* < 0.001; d = 1.07; pre vs. 30 min post-exercise: mean difference = −4.04 nmol/L *p* < 0.001; d = 1.58).

Cortisol values (nmol/l) were significantly higher at all time points in the pubertal group vs. the prepubertal group, except for the baseline levels (*p* = 0.157). A significant main effect for time (*p* = 0.032, *η*^2^ = 0.231) and group (*p* = 0.010, *η*^2^ = 0.175) was observed. However, no significant time-by-group interaction effect was found (*p* = 0.212; *η*^2^ = 0.126). Pairwise comparisons showed no significant acute effects in cortisol levels for any time points or group (*p* > 0.05).

There was a significant main effect for time (*p* = 0.011, *η*^2^ = 0.278) and group (*p* < 0.001, *η*^2^ = 0.487) in SHBG, but no time-by-group interaction effect (*p* = 0.869, *η*^2^ = 0.001). Pairwise comparisons showed no significant acute effects in SHBG levels for any time points or group (*p* > 0.05).

Free androgen index showed a significant time (*p* < 0.001, *η*^2^ = 0.538) and time-by-group interaction effect (*p* < 0.001, *η*^2^ = 0.541). Follow-up pairwise comparisons showed no acute effect in the free androgen index for the prepubertal group but a significant decrease following the resistance training session at 15 min (*p* < 0.001; d = 0.80) and 30 min (*p* < 0.001; d = 1.14) post-exercise for the pubertal group.

### Performance in acute resistance training

**Fig. 3:**
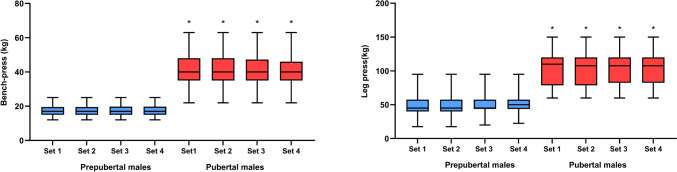
Median load lifted during the acute resistance training program in bench press and leg-press exercises separated by groups. ^#^Significantly different between prepubertal and pubertal group; *p* < 0.05

The prepubertal and pubertal groups maintained the exercise load across all four sets of bench press and leg press exercises. The average load lifted during the acute resistance training program (Fig. 3) was significantly (*p* < 0.05) greater in the pubertal group vs. prepubertal group, in both bench press (40.5 ± 10.0 kg vs. 17.5 ± 3.7 kg) and leg press exercise (103.8 ± 24.8 kg vs. 50.2). The average heart rate during the resistance training program was 136 beats/min (SD: 17.1) for prepubertal males and 143 beats/min (SD: 16.4) for pubertal males (*p* = 0.014). The average rating of perceived exertion throughout the program was similar between the groups, 8/10 for prepubertal males and 9/10 for pubertal males. Although the load was constant, the repetitions completed varied depending on the group, which was evident in the calculation of fatigue index. Prepubertal males had a lower fatigue index than pubertal males in the bench press exercise (27.6 ± 25.9% vs. 58.9 ± 31.8%; *p* = 0.01, η^2^ = 0.234), but no significant difference was observed in the leg press exercise (2.4 ± 8.9% vs. 0.5 ± 2.2%; *p* = 0.364, η^2^ = 0.021).

## Discussion

The present study showed that testosterone and IGF-I responses following an acute bout of free-weight resistance training differ between trained prepubertal and pubertal male children. The main finding was that an intense bout of moderate-intensity free-weight resistance training conducted to failure led to increased concentrations of anabolic mediators (IGF-I, testosterone) in pubertal male children but not in prepubertal. In contrast, in the prepubertal children, but not the pubertal, we found increased IL-6 levels, indicating a stronger pro-inflammatory response after the acute resistance training session.

### GH-IGF-I axis* response to resistance training*

In the current study, we observed a large increase in post-exercise GH levels after the resistance training session that returned to baseline at post 30-min for both the prepubertal and pubertal children. The peak GH levels were higher in the pubertal group compared to the prepubertal group. Our results are in line with one other study on resistance training, showing a post-exercise GH increase in children following resistance training (Rubin et al. 2014), while another study showed no significant increase in post-exercise GH response in children (Pullinen et al. 2011). The magnitude of GH increase in our data is somewhat larger/higher compared to what was observed in the previously mentioned studies that used lighter exercise protocols such as weighted step-up exercises and bilateral knee extensions (Pullinen et al. 2011; Rubin et al. 2014). As it is known that training intensity is a key factor causing increased resistance training-induced GH response in adults (Kraemer and Ratamess 2005), one can speculate that this is also true for children. High training intensity (e.g., high levels of lactate) typically induces greater GH response to resistance training (Kraemer and Ratamess 2005). In our data, the pubertal group had a higher peak GH response to resistance training compared to the prepubertal group. Since children have lower post-exercise lactate levels and activate their type II motor units to a lesser degree than adults (Dotan et al. 2012), an overall lower peak GH response to exercise is expected even at higher training loads in prepubertal compared to pubertal children. Our results showed a significant difference in IGF-I levels between prepubertal and pubertal children, primarily due to growth. Our results showed a moderate acute increase in levels of IGF-I immediately post-exercise in the pubertal group but not in the prepubertal group. Previous studies examining the IGF-I response to resistance training in children and adolescents are scarce (Falk and Eliakim 2014; Rubin et al. 2014). In contrast to our results, *Rubin *et al. found a significant post-exercise increase in IGF-I in both 9-year-old children and adults despite using modest intensity resistance training (step-up with weight west) (Rubin et al. 2014). Moreover, the pre-post increases in IGF-I were greater in the same study, compared to our data (15% vs. 6%) (Rubin et al. 2014), although we adopted a training program with a higher load.

Training load has been suggested as an important factor for the IGF-I response to exercise (Eliakim and Nemet 2012). Exercise training is expected to result in an increased acute IGF-I response. However, other studies conducted in children and adolescents with longer training duration (90 min) and hence higher training load have shown decreased IGF-I levels post-exercise after a single session of intense anaerobic (Nemet et al. 2002) and aerobic training (Nemet et al. 2003). Differences in training load between our study and Rubin et al. (2014) may explain the different IGF-I responses. The relatively short, modest-intensity protocol adopted in the study by Rubin et al. (2014) significantly increased IGF-I post-exercise response, even in 9-year-old children. Hence, the high workload applied in our study may have damped the overall IGF-I response, in particular for the prepubertal group. These results may suggest that exercise load should be adapted based on maturation and possibly that a lower overall training load should be used when designing resistance training programs for prepubertal compared to pubertal children. Future longitudinal studies are needed to examine the impact of resistance training load in children.

### Cytokine response to resistance training

There were significant differences in TNF-α and IL-6 levels between the prepubertal and pubertal groups, with baseline values indicating that growth affects cytokine levels. Additionally, a significant acute increase in TNF-α levels was observed in both groups following resistance training. However, only the prepubertal group showed an increased IL-6 response post-exercise. The effect sizes increased from a moderate effect at the post and 15-min post exercise to a large effect at 30-min post-exercise. Previous research has suggested that the proinflammatory cytokines (IL-6 and TNF-α) partly contribute to the reductions in anabolic mediators typically observed after an initial training program in youth (Nemet et al. 2002). This has almost exclusively been examined in endurance or sports-specific activities, not in resistance training (Nemet et al. 2002, 2003). Hence, it seems as though resistance training, similar to endurance training, results in a simultaneous increase in circulating levels of cytokines. This is supported by a study that examined the response after a single anaerobic-type training session (wrestling session) that showed large post-exercise increases in cytokines IL-6 and TNF-α in adolescent males (Nemet et al. 2002).

Interestingly, our data showed increased levels of TNF-α in both groups after the resistance training session, but only a significant increase in IL-6 in the prepubertal group, accompanied by a small decrease or no change in IGF-I. Conversely, the pubertal group showed no significant increase in IL-6 but experienced a moderate rise in IGF-I following the exercise protocol. The IL-6 acute response reported in our data was smaller than what has been seen in other studies in youth (Nemet et al. 2002, 2003). Based on adult studies, the magnitude of the exercise-induced IL-6 response depends on the intensity and especially the exercise duration, while the exercise mode has little effect (Fischer 2006). IL-6 is typically released from the muscle due to low glycogen (Fischer 2006). Hence, one can speculate that our study’s training duration was too low to induce large IL-6 responses compared to previous pediatric studies that typically adopt longer (1.5 h) training durations (Nemet et al. 2002, 2003).

In summary, the high workload applied in our study may have caused the increased levels of pro-inflammatory cytokines, which may have damped the IGF-I response. Hence, this may explain the overall smaller acute response in IGF-I following resistance training in our study compared to Rubin et al. (2014). Taken together, it is possible that the moderate resistance training session conducted to failure was too intense for the prepubertal children in our study, causing a moderate- to large IL-6 response and suppression of the GH-IGF axis. Hence, our data may partly support previous studies suggesting cytokines may inhibit the acute IGF-I response of youth (Nemet et al. 2002). However, we report large variations in IL-6 and smaller changes than evident in previous research, which should be taken into consideration (Nemet et al. 2002, 2003).

### Testosterone and cortisol response to resistance training

As expected, our data showed a significant difference in testosterone levels between the prepubertal and pubertal groups. The pubertal group had higher basal testosterone levels and a greater acute pre-post decreases in response to resistance training compared to the prepubertal group. Our results are in line with another study that showed a significant difference in the acute testosterone response following body-weight resistance training between young males aged 12–13 versus 18-year-old males (Sekine and Hirose 2022). This suggests that resistance-induced acute testosterone response may depend on maturity status.

Testosterone levels increase with puberty and correlate to muscular strength levels in children and adolescents (Round et al. 1999). Other factors that have been reported to influence the acute testosterone response to resistance exercise include muscle mass, intensity, training volume, and training experience (Kraemer and Ratamess 2005). The intensity of the training session in our study was constant between the groups (e.g., 10 RM), and all participants reported having little resistance training experience. Hence, differences in growth (e.g., pubertal group greater LLV and FFM) between the groups are probably important for the acute testosterone response. The larger acute response we observed in the pubertal group compared to the prepubertal group may further be due to the prepubertal’s smaller testicular volume and fewer or less differentiated Leydig cells (Falk and Eliakim 2014). Many of these factors (e.g. FFM, LLV, testicular volume) are associated with maturity status.

Our study showed a large acute decrease in testosterone levels at 15 min and 30-min post-exercise in the pubertal group, while no acute effect of training was observed in the prepubertal group. Similarly, free androgen index decreased only in the pubertal group (15-min and 30 min post-exercise) but no change in the prepubertal group, Previous studies have focused on adolescents and early adolescents and typically show small acute testosterone increases, which are normalized shortly after resistance exercise (Kraemer et al. 1992; Pullinen et al. 1998, 2002; Falk and Eliakim 2014; Sekine and Hirose 2022). In contrast, our data on pubertal males showed a significant post-exercise decrease in testosterone, possibly explained by the intense training program adopted in the study (average rating of perceived exertion > 8). In support, a study conducted on male Taekwondo athletes (12–17 years old) also reported a decreased post-exercise testosterone response and no change in SHBG following an intense protocol, including three consecutive matches (Pilz-Burstein et al. 2010). The authors interpreted the response as a catabolic-type hormonal response (Pilz-Burstein et al. 2010). Similarly, our data showed no change in SHBG, and thus, our data supports the theory that it may be the catabolic-type response.

Furthermore, our data showed no significant differences within or between groups in post-exercise increases in cortisol. The acute cortisol response to resistance training in adolescents typically increases following resistance training, but whether it is affected by age or maturation is less clear (Falk and Eliakim 2014). Pullinen et al. (2011) reported higher post-exercise cortisol values in adults compared to adolescents following bilateral knee extensions until exhaustion. Previous studies have suggested that the initial rise in testosterone is inhibited by an increase in cortisol (Cadoux-Hudson et al. 1985), which may explain why the patterns of cortisol seem to be different between groups in our data (e.g., testosterone decrease and cortisol increase in the pubertal group).

### Performance during exercise

Large significant differences in exercise-induced fatigue were observed between the prepubertal and pubertal groups during the leg press exercise, but not in the bench press exercise. The pubertal group showed a greater decline in repetitions completed at a 10 RM load in the bench press compared to the prepubertal group. Exercise-induced fatigue was evident in both groups following the training protocol, reflecting the exhausting nature of the protocol with large muscle mass involved. Large differences in loads lifted were also seen between the groups, showing the effects of growth on muscular strength. These data are important findings since a previous systematic review and meta-analysis (Souron et al. 2022) have highlighted that the current literature has rarely considered the adolescent versus children comparison for the evaluation of fatigue. Similar studies support our data showing that maturation affects fatigue (Pullinen et al. 2002; Tibana et al. 2012). Tibana et al. (2012) reported a higher recovery capacity between sets of resistance training in 15-year-old adolescents compared to adults (Tibana et al. 2012). Similarly, Pullinen et al. (2002) evaluated the exercise-induced fatigue in an isometric maximal voluntary contraction (MVC) test of the knee extensor muscles and reported greater fatigue in the adult vs. the adolescent group. Our data showed small group differences in exercise-induced fatigue in the leg-press test, most likely due to inappropriate equipment unsuited for repeated 10RM sets. Hence, data from the leg-press exercise should be interpreted with caution. Overall, these data suggest that prepubertal children have a greater ability to resist fatigue during resistance training compared to pubertal children, which implies that shorter inter-set rest periods may be used when designing resistance training programs for prepubertal children.

### Strengths and limitations

This is the first study, to our knowledge, that has evaluated the acute hormonal and cytokine response to free-weight resistance training according to pubertal stage in male children. The main strength are the rigorous study design with a 3-day test session, with individually programmed exercise intensity, designed to habituate participants to the adopted training session and technique of the exercises. Furthermore, the study investigated several hormonal and cytokine responses to resistance training, which has been a knowledge gap, particularly for the prepubertal child population. In addition, we used free-weight resistance training that are commonly used in actual field practice, including both upper- and lower-body exercises well-suited for examining acute hormonal response. The study’s main limitation is its relatively small sample size, as well as its only inclusion of youth male athletes. Generalizations regarding untrained youth still need to be examined. Additionally, this study did not strictly control participants’ caloric intake prior to exercise, which might have had some influence on the hormonal responses observed. Future studies could benefit from implementing more rigorous dietary controls to further clarify this aspect. Lastly, no females were included in the study, so careful interpolation of the female population should be made. Hence, we suggest future studies to explore the acute effects of resistance training on hormones and proinflammatory cytokine in a female child and adolescent population.

## Conclusion

The main finding from this study showed that the hormonal and cytokine response following an acute bout of free-weight resistance training differs between trained prepubertal and pubertal male children. Mature children had a more pronounced post-exercise hormonal response (testosterone and IGF-I) than their less mature peers after a moderate-intensity free-weight resistance training session conducted to failure. However, for young prepubertal male children, we found a more pronounced proinflammatory response that may have suppressed the GH-IGF-I axis. Acute neuromuscular performance decrements were observed in prepubertal and pubertal male children, but the prepubertal child group had a better ability to resist fatigue. These findings suggest that while the training protocol regarding hormonal responses may have been overly strenuous for prepubertal male children, it was appropriately targeted to induce hormonal adaptations in pubertal male children. Hence, training load may be an important variable when designing resistance training programs based on maturation. Larger cohort studies are needed to confirm the results, and future longitudinal studies are needed to investigate the chronic implications of these acute responses.

## Data Availability

The datasets generated during and analyzed during the current study are available from the corresponding author upon reasonable request.
